# Correlation between mobility in mass transport and mortality due to COVID-19: A comparison of Mexico City, New York, and Madrid from a data science perspective

**DOI:** 10.1371/journal.pone.0264713

**Published:** 2022-03-17

**Authors:** Andrea Vega-Villalobos, Nelva Nely Almanza-Ortega, Kirvis Torres-Poveda, Joaquín Pérez-Ortega, Igor Barahona

**Affiliations:** 1 Tecnológico Nacional de México/CENIDET, Cuernavaca, Morelos, México; 2 Tecnológico Nacional de México/IT de Tlalnepantla, Tlalnepantla de Baz, Estado de México, México; 3 Centro de Investigación Sobre Enfermedades Infecciosas, Instituto Nacional de Salud Pública, Cuernavaca, Morelos, México; 4 CONACyT-Instituto Nacional de Salud Pública, Cuernavaca, Morelos, México; 5 Universidad Nacional Autónoma de México, Instituto de Matemáticas, Laboratorio de Aplicaciones de las Matemáticas, Cuernavaca, Morelos, México; Nanyang Technological University, SINGAPORE

## Abstract

In most big cities, public transports are enclosed and crowded spaces. Therefore, they are considered as one of the most important triggers of COVID-19 spread. Most of the existing research related to the mobility of people and COVID-19 spread is focused on investigating highly frequented paths by analyzing data collected from mobile devices, which mainly refer to geo-positioning records. In contrast, this paper tackles the problem by studying mass mobility. The relations between daily mobility on public transport (subway or metro) in three big cities and mortality due to COVID-19 are investigated. Data collected for these purposes come from official sources, such as the web pages of the cities’ local governments. To provide a systematic framework, we applied the IBM Foundational Methodology for Data Science to the epidemiological domain of this paper. Our analysis consists of moving averages with a moving window equal to seven days so as to avoid bias due to weekly tendencies. Among the main findings of this work are: a) New York City and Madrid show similar distribution on studied variables, which resemble a Gauss bell, in contrast to Mexico City, and b) Non-pharmaceutical interventions don’t bring immediate results, and reductions to the number of deaths due to COVID are observed after a certain number of days. This paper yields partial evidence for assessing the effectiveness of public policies in mitigating the COVID-19 pandemic.

## Introduction

Coronavirus disease 2019 (COVID-19) continues to devastate many communities and economies, placing healthcare systems under mass pressures. Strategies implemented in order to mitigate this disease include various Non-Pharmacological Interventions (NPIs) such as travel restrictions, curfews, lockdowns, social distancing regulations, and tracking of positive contacts [1]. Depending on the level of contagion in different countries, various strategies combining different NPI measures have been used [1, 2]. Among them are the use of rapid tests together with the prompt isolation of suspected cases, confirmed cases, and their contacts. In [3], sanitary cordons that confined bigger populations into specific geographic areas were documented. Other papers, such as [4] documented types of interventions aimed to reduce human mobility, including school and university closures, restrictions on meetings and gatherings, closures of all economic activities considered non-essential, and even border closures.

A systematic review of empirical studies found that school closings, followed by workplace closings, business and venue closings, and public-event bans were the most effective NPIs controlling the spread of COVID-19 [5]. [6] reported that restrictions of 90% to travel to and from mainland China only modestly affected the epidemic trajectory unless combined with a 50% or higher reduction of transmission in the community [6].

Human mobility patterns have been used to investigate how pandemics are propagated and how contagion rates can be mitigated [7, 8]; this is also the case for COVID-19 [3]. In the particular case of COVID-19, mobility patterns constitute one of the most important variables predicting the number of deaths due to this disease [9].

It has been proposed that mobility data can serve as a proxy for social contacts [10]. The use of mobility measures can reflect the level of contact and therefore the level of transmission [10–12]. Recent research has shown that social mobility plays a significant role in the transmission dynamic [10, 13]. The correlation between different mobility indices and new COVID-19 cases in different Portuguese districts has been reported [13], and it has been shown that mobility in retail and recreation, grocery and pharmacy, and public transport has a higher correlation with new COVID-19 cases than mobility in parks, workplaces, or residences.

The transmission of respiratory diseases, such as SARS-Cov-2, from one infected person to another susceptible person, mostly indoors, has been widely documented in the literature [14]. Public transport, such as subways or metros, allows passengers to enter in close contact with infected people and breathe the virus, even if the infected passenger never coughs or sneezes. Infected persons constantly contaminate many surfaces they come in contact with, such as glasses, keys, and telephones. This means public transportation offers COVID-19 an efficient means of propagation. The above makes this type of public transport an ideal and efficient way for SARS-Cov-2 transmission [15].

In crowded cities with high population densities, public transport is believed to be among the most important sources of COVID-19 contagion. The associations between traveling by train, subway, or metro and contagion rates have been documented [16], and the association between the load of domestic passengers (daily numbers of passengers) departing from Wuhan to the city clusters of each province and the number of confirmed cases exported to the 10 city clusters in mainland China have been evaluated. This study found a statistically significant positive association between travel by train and spread of COVID-19 infections outside Wuhan [17]. In addition, in the study of [17], a positive association was found between the frequency of flights, trains, and buses from Wuhan and the daily and accumulative numbers of COVID-19 cases in other cities, with progressively increased correlations for trains and buses [18]. A reduction in transmission has been explicitly linked to the reduction in mobility [19, 20], and reductions in population mobility can mitigate disease-related mortality [21].

In this context, the main objective of this paper is to investigate the correlation between the daily mobility of those who use public transport (subway) and mortality due to COVID-19. We present a case study using official data sources from the following big cities with similarities in population or subway system: New York City (NYC), Madrid (MAD), and Mexico City (CDMX), thus allowing a unique opportunity to compare similar cities in different contexts.

## Materials and methods

This research is based on the methodology proposed by IBM Inc. [22]. It primarily focuses on designing and developing Data Science projects. One original contribution of this paper is extending the first stage of the aforementioned methodology, with an orientation to the epidemiological domain. For this extension, basic concepts of epidemiology were integrated and merged with particularities of data science, which contributes to a systematized process that allows for a better understanding of the COVID-19 pandemic. This extension addresses the following question: Are there correlations between mobility through public transport and death due to COVID-19?

In order to answer this question, we selected three main cities: CDMX, MAD, and NYC. Among the main reasons for selecting these cities are similar population size and distribution, which is greater than 6 million and with an average age of 35–41 years; both CDMX and NYC had daily traffic of approximately five million users prior to the pandemic and accessibility to daily-mobility data and COVID-19 indices. The sources from which data were collected are listed below:

- New positive cases and deaths in CDMX [23].
- Daily trips reported for the entire CDMX metro network [24].
- New positive cases and deaths per day in NYC [25].
- Daily trips reported for the entire NYC subway network [26].
- New positive cases and deaths in MAD [27].
- Daily trips reported for the entire MAD metro network [28].

There are some important terms related to the data: 1) **Positive cases** are given by the number of people who were confirmed positive daily by a SARS-Cov-2 laboratory test, 2) **Deaths** represent the number of daily deaths as a direct consequence of being infected by SARS-Cov-2, and 3) **Mobility** is given by the number of daily users of public transport and expressed as the total number of trips per day, taking into account that one person might take more than one trip per day. Each trip begins when a user enters a station and ends when that user exits at a different one, regardless of the number of connections. For this research, only trips by rail, subway, or metro are considered.

Note that activities related to collecting data on daily trips were carried out differently across the investigated cities. First, NYC began to publish mobility data on March 1st, 2020. Second, the number of trips is updated daily in NYC, whereas monthly updates are carried out in MAD. Finally, updates are performed irregularly in CDMX, the last of which was done on October 31, 2020. This time window delimits the framework of our research. A preliminary analysis of available data allowed us to identify patterns on collecting and registration processes, which might be associated with weekdays. For example, the lowest values of the investigated variables occurred on weekends in contrast to weekdays. To overcome this issue, Moving Averages (MA) were calculated. MA are helpful tools for comparisons between time series. They are given by the average of *m* observations. In this research, the moving average for day *t* corresponds to the average of *x*_*j*_ observations of cases, mortality, or mobility in a period that starts on day *t* -(*m*-1) and ends on day *t*.

While the main purpose of MA is to capture the main trend of data by reducing fluctuations, they are suitable for contrasting time series from different groups or events (e.g., cities). The order of MA is given by the window size, and it also determines its degree of smoothness. According to [29], the seasonal effect on a time series is the most important aspect to consider when the optimal size of the windows is determined. In this work we experimented with several values of *m* and found that the best value was *m* = 7 to represent weekly data. Below in Eq 1 is the definiton for MA, denoted by ${\hat{P}}_{t}$.

$$\begin{array}{c} {\hat{P}}_{t}=\frac{1}{m}\sum_{j=t-(m-1)}^{t}x_{j} \end{array}$$ (1)

Where:

${\hat{P}}_{t}$ is the MA given *m* value at day *t*;

*x*_*j*_ represents the value for day *j*;

*m* represents the value of the mobile window.

While comparing cities for each time series, observations belonging to the same window are more likely to be closer in value. Based on the above, MA are able to reduce randomness by preserving a smooth trend-cycle component.

Prior to the analysis, three types of normalization were applied to our datasets: a) the normalization min-max scaler (MMS), b) a novel Composite Indicator, called *ψ*, and c) the Cross-Correlation Function (CCF).

MMS allows for data comparisons across cities, makes sense, since this type of standardization yields values within a range [0,1]. The mathematical expression for MMS is shown in Eq 2.

$$\begin{array}{c} x_{t}=\frac{x-min(x)}{max(x)-min(x)} \end{array}$$ (2)

Where:

*x*_*t*_ is the standardized value of the variable of interest for a given day *t*;

*x* represents the observed value for a given day;

*min*(*x*) is the minimal value of the period;

*max*(*x*) is the maximum value of the period.

A new composite indicator (CI), called *ψ*, is proposed. In order to quantitatively compare the effectiveness of pandemic control and mitigation measures in a city, expressed as the number of deaths in a day, relative to the number of trips in the city’s subway on the same day. According to [30], a CI *ψ* is given by a combination of two or more indexes. It is a helpful tool for describing multidimensional problems or abstract concepts, as is the case of relations between mortality due to COVID-19 and urban mobility. Other examples of abstract concepts are economic growth, happiness, or well-being. CI *ψ* is defined by Eq 3:

$$\begin{array}{c} \psi_{t}=\frac{\sum_{j=t-(m-1)}^{t}d_{j}}{\sum_{j=t-(m-1)}^{t}m_{j}}=\frac{{\hat{D}}_{t}}{{\hat{M}}_{t}} \end{array}$$ (3)

In Eq 3, ${\hat{D}}_{t}$ represents the MA of the number of deaths due to COVID-19 for day *t* and ${\hat{M}}_{t}$ denotes the MA for the mobility index for a given day *t*. The observation of deaths for day *j* in a city is represented by *d*_*j*_ and mobility by *m*_*j*_.

CI *ψ* has two interesting properties: a) It is independent of the population size of the cities, and b) It is independent of the capacity of the subway system. Those properties allow for quantitative comparisons between cities with different population size and metro system capacity. The city with the lowest value of *ψ* denotes a more effective pandemic management.

**Fig 1:**
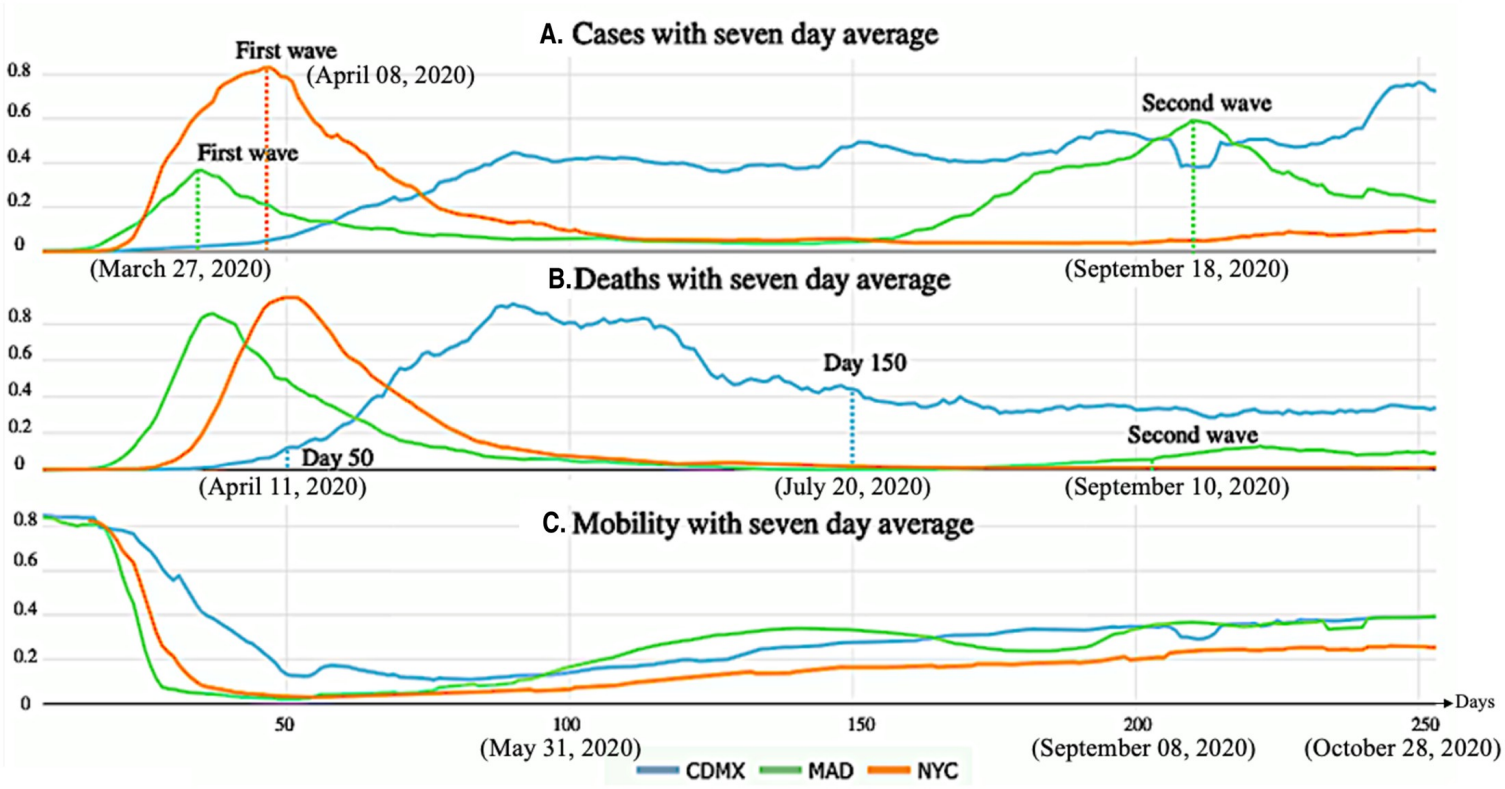
Seven-day average. Section A: MA with *m* = 7 for positives cases. Section B: MA with *m* = 7 for deaths due to COVID-19. Section C: MA with *m* = 7 for mobility index.

**Fig 2:**
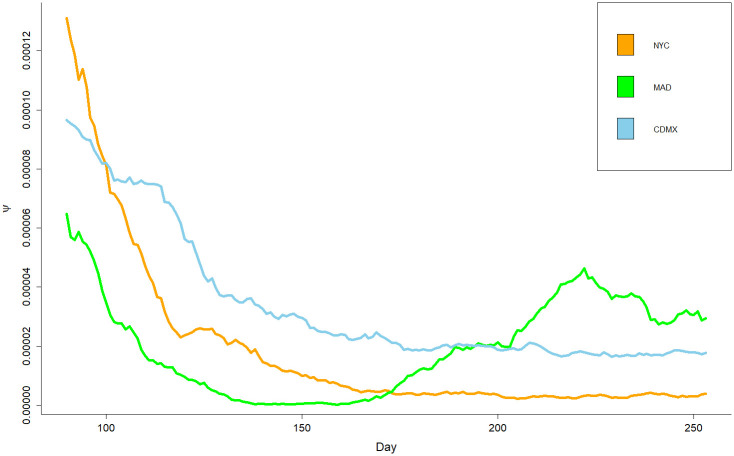
Visual representation of indicator *ψ* for the investigated period, from May 21st to October 31st, 2020.

In this research, in order to properly interpret the changes in *ψ* values or trends, the following facts are assumed: a) In general, $\hat{M}$ (mobility) values were at their lowest before day 100 of the pandemic and continued to increase thereafter (see Fig 1C and 1b) $\hat{D}$ (mortality) values are significantly lower than $\hat{M}$ values by several orders of magnitude. Based on the above facts it can be stated, in general, that changes in *ψ* values are more attributable to changes in $\hat{D}$ than to changes in $\hat{M}$. An example of this is the noticeable changes in *ψ* values in the city of Madrid, which occurred after day 150 of the pandemic (see Figs 1A and 2).

The Cross Correlation Function (CCF, denoted by $\rho_{\hat{D}\hat{M}}$) is a well-known method to assess the degree of similarity between two sets of numbers (e.g., time series). From another perspective, CCF can be approached as the dot product of two vectors $\vec{m}$ and $\vec{d}$. Although CCF is a simple concept, it generally constitutes the basis for more advanced analysis. The normalized CCF is obtained by subtracting the average on time series ${\hat{D}}_{t}$ and ${\hat{M}}_{t}$ and dividing it by the product of standard deviations $\sigma_{\hat{D}}$ and $\sigma_{\hat{M}}$, where ${\hat{D}}_{t}$ denotes the time series for the number of deaths due to COVID-19 and ${\hat{M}}_{t}$ is the time series for mobility index. Note that these series are standardized within [0–1] range. $\sigma_{\hat{D}}$ and $\sigma_{\hat{M}}$ are the standard deviations for the mentioned time series. Considering CCF is limited to the range $-1\leq \rho_{\hat{D}\hat{M}}\leq 1$, negative values denote an inverse relation; on the other hand, positive values represent a direct one. A formal definition of CCF is given by Eq 4.

$$\begin{array}{c} \rho_{\hat{D}\hat{M}}=\frac{\sum_{i=0}^{n-1}\left(d_{i}-\bar{d}\right)*\left(m_{i-l}-\bar{m}\right)}{\sqrt{\sum_{i=0}^{n-1}{\left(d_{i}-\bar{d}\right)}^{2}}*\sqrt{\sum_{i=0}^{n-1}{\left(m_{i}-\bar{m}\right)}^{2}}} \end{array}$$ (4)

From a statistical perspective, Eq 4 is known as the Pearson product-moment correlation coefficient [31]. It is interpreted as the extent to what time series ${\hat{D}}_{t}$ and ${\hat{M}}_{t}$ vary from one another, divided by the amount of its variability degree separately. According to [32], CCF should not be calculated for values of *l* close to *n*, and therefore l < *n*. They also suggest a practical empirical rule for calculating *l* values, where *l* should be smaller than *n*/2. In this paper, CCFs for mortality (${\hat{D}}_{t}$) and mobility (${\hat{M}}_{t}$) were calculated for windows within a range -21 < l < 21, as shown in the following section.

## Results

This section describes the results divided into three aspects. First, a study on the behavior of the pandemic in terms of cases, deaths and mobility; second, the implementation of the proposed CI *ψ* highlighting the period for days 90 to 253, in which it is possible to observe the different trends for each of the cities. Finally, the results of the CCF between daily deaths and mobility are shown for each city.

Moving averages, in a range [0, 1], for positives cases, deaths due to COVID-19, and mobility index in Fig 1A–1C are respectively shown. Notice that Eq 2 was applied to normalize the data, then MA were calculated by using Eq 1 with the parameter *m* = 7. Investigated cities are presented in each figure with blue, green, and orange colors for Mexico City (CDMX), Madrid (MAD), and New York City (NYC), respectively. While the horizontal axis is the same for the figures with *n* = 253 days, values for the three indicators mentioned are displayed on vertical axes. For Fig 1A, whereas the first peaks are reached on March 27th and April 8th, 2020, in MAD and NYC, respectively, a second spike occurred in MAD on September 18th, 2020. It is important to notice the differences in the curve shape for MAD on the one hand, and the curve shapes for NYC and CDMX on the other. While the curve for MAD is clearly bimodal, NYC shows a unimodal curve and CDMX presents a constantly increasing pattern.

Regarding Fig 1B, it is striking that approximately 80% of the deaths for CDMX (blue curve) took place between April 11th and July 20th, 2020. There is a soft spike on September 10th, 2020, for MAD, which indicates a slight second wave in this index. Peaks in positive cases and deaths almost match for NYC; while the former took place on April 8th, the peak for deaths was on April 11th, 2020. Whereas death curves for MAD and NYC show similar patterns, there are evident differences with respect to the CDMX curve. An important reduction in the mobility index in Fig 1C is observed, mainly due to NPIs implemented by authorities in the three investigated cities.

Eq 3 models the relation between mortality and mobility. In Fig 2, on the *y* axis the values of *ψ* are shown, and on the *x* axis the days of the interval studied [90–253]. Fig 2 shows the values of indicator *ψ* expressed by three continuous lines. Each line is shown with a different color for each city: New York (NYC) is shown in orange, Madrid (MAD) is depicted in green, and Mexico City (CDMX) is shown in blue. Next, the behavior of *ψ* for the three cities is described.

On day 90, NYC has the highest values of *ψ*; however, the subsequent days show a negative slope of the line and an inflection on day 132 approximately. Notice that from day 150, NYC has an almost constant value of *ψ*. And for the interval of the days 170 to 253 approximately, NYC has the lowest values of *ψ*, which suggests that for this interval the handling of the pandemics was more effective in NYC than in the rest of the cities.

In the case of MAD, the values of *ψ* were smaller than those of other cities between days 90 and 160. After day 160 until day 225, the values of *ψ* increase significantly due to an increase in mortality. It is known that MAD relaxed its NPIs measures after the first pandemic wave, which suggests that this made possible the second wave. Fig 4 shows that MAD ended the emergency state in June and reopened multiple accesses to the subway.

CDMX shows values of *ψ* similar to those of NYC, but on a larger scale. Only the values of *ψ* for MAD are larger than those of other cities during its second wave.

**Fig 3:**
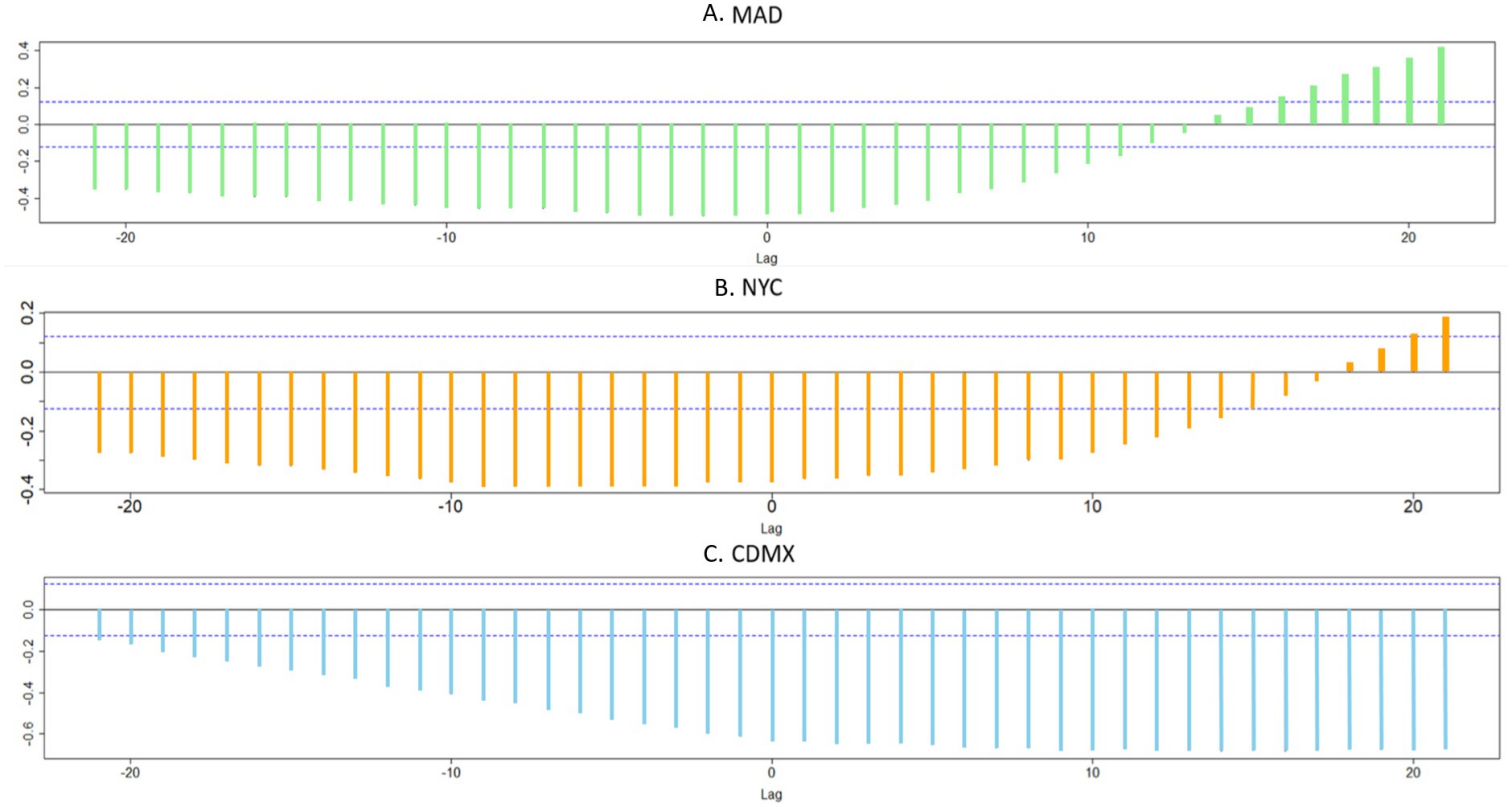
CCF between daily deaths and mobility for each city.

CCF values for MAD, NYC, and CDMX are respectively shown in Fig 3A–3C. Note that CCF values are within [-1,1] range, and calculated on the basis of Eq 4. In order to calculate precise CCF estimations, an optimal mobile-window size should be used. The above avoids losing patterns that are relevant to the investigated problem due to a very short mobile-window size. On the other hand, a very large mobile window might add irrelevant or noisy information. The criteria proposed by [29] was adopted for these purposes. Thus, the window size for our CCF estimation is equal to ±√*n*+6, where *n* = 253 represents the total number of observations (days) included in the study. On the one hand, vertical lines represent CCF values at different lag times with a window size equal to ±√253+6=11. On the other, vertical dotted blue lines refer to the hypothesis testing for different CCF values. We are testing *H*_0_: there is statistical evidence to ascertain that CCF is different from zero ($\rho_{\hat{D}\hat{M}}\left(t\right)\neq 0$) at time *t*, with a level of significance equal to *α* = 0.05. Therefore, vertical lines falling beyond the vertical dotted blue line represent CCF values that are statistically significant at a 0.95 confidence level. Due to space limitations, the way threshold values for hypothesis testing were calculated has been omitted. Readers are encouraged to consult [29] for a detailed explanation of CCF.

Fig 3A–3C yields empirical evidence to assess the impact of NPIs on the COVID-19 pandemic. Negative values of CCF are observed in Fig 3A for MAD until lag *l* = 13, then a sign change occurs at *l* = 14. This point (*l* = 14) corresponds to April 16th, 2020. Based on the above, a direct relation between mortality ${\hat{D}}_{t}$ and mobility ${\hat{M}}_{t}$ starts at *l* = 14, which suggests that the impact of NPIs on reducing the number of deaths due to COVID does not produce immediate results. For NYC, the sign change occurs at *l* = 18, which corresponds to April 20th, 2020. The above suggest that NPIs were more effective in MAD than those in NYC, yielding results in a shorter period. On the other hand, there is no evidence that NPIs yielded positive results in CDMX, considering that a correlation between deaths and mobility is not positive along the investigated period.

## Discussion

The results of this study, through a methodology that combines the most relevant aspects of epidemiology within a Data Science framework, provide valuable information about relations between public transport mobility and mortality due to COVID-19. Additionally, the use of the methodology allows establishing a direct connection between the characteristics of the research question and the selection of various solution methods.

CDMX showed different patterns compared to the other cities. In this city, a clear pattern of mortality was identified and the mortality rate declined at the same time as the index of mobility declined. As shown in Fig 1B, the highest values for mortality in CDMX were reached during the period from days 71 to 129, which correspond to the period from May 2nd, 2020, to June 29th, 2020, even with the corresponding reduction in mobility index over the same period.

A key aspect to consider for investigating the effect of mobility on the death rate from COVID-19 is the analysis of the NPIs implemented by different governments around the world. A previous study showed that NPIs are effective for reducing the contagion rates and, consequently, flattening the curve of accumulated cases [33]. Other studies calculated to what extent the NPIs were effective in European cities, either individually [2, 34, 35] or generally [4]. Both groups of studies confirmed that NPIs are effective in mitigating contagion rates.

**Fig 4:**
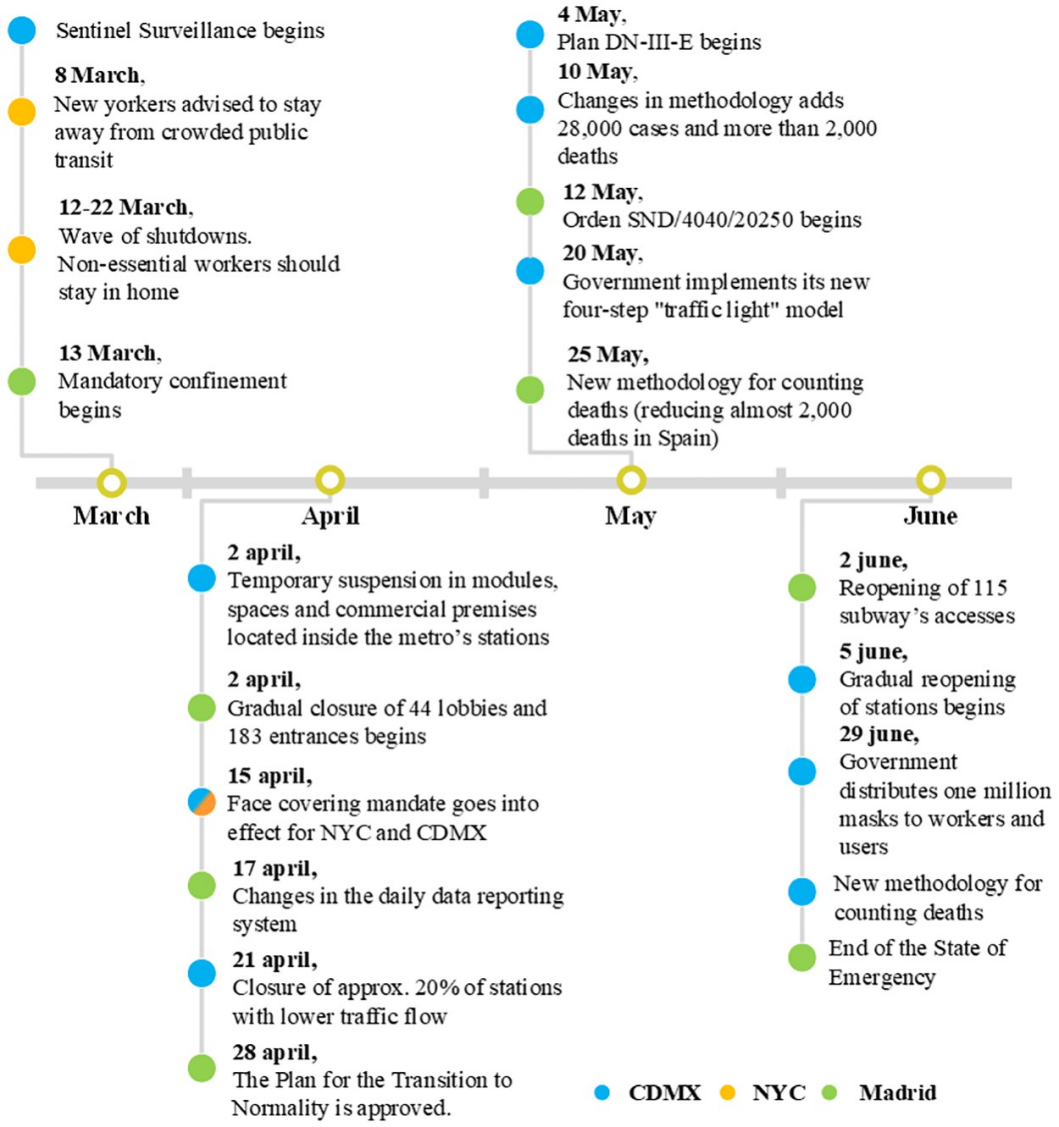
NPIs timeline. Timeline of different NPIs and methodologies applied in the studied cities during the first months of the COVID-19 pandemic.

The relation between each NPIs time ranges and changes in the internal measures of rail-trip system operation in CDMX, MAD, and NYC are presented in Fig 4. In this sense, CDMX applied less restrictive mobility regulations for shorter periods in contrast to MAD and NYC. The mobility dynamic and related sociodemographic variables are among the main causes of these differences. Notice that all countries carried out methodological changes for collecting and registering data. A methodology called Sentinel Surveillance was implemented at the very beginning in CDMX, followed by two important changes. The first, on May 10th, 2020, consisted of adjustments to the way deaths were counted. The second was in June, when a total of 28,000 positive cases and almost 2,800 deaths were added to the national statistics. MAD, on April 28th, 2020, implemented a change to the system for collecting daily data; later, on May 25th, 2020, a decrease of approximately 2,000 deaths was reported because of a change of methodology.

Subway or metro systems and networks of the three cities included in this study show different degrees of heterogeneity in regards to designs, technologies, and capabilities. For instance, there are subway system networks with elevated stations (above ground level) in MAD or CDMX, whereas the NYC subway system network is mainly underground. There are systems focused on providing only local transportation, such as the NYC subway, while others can offer transportation across the city, as the MAD or CDMX systems do [15].

Most NPIs generate significant costs for societies [4]. For low- or middle-income countries, with important income inequalities and a significant sector of the population working in an informal economy, such as Mexico [36], NPIs brought devastating consequences: increasing unemployment [37, 38], crime [39], and domestic violence [40]. Many self-employed people belonging to the informal economy have been incapable of completely following NPIs, and they have been kept exposing themselves despite the epidemiological recommendations of social distancing [41]. The peak in the daily number of positive cases was reached in CDMX, at the time metro services were reduced by local governments. As suggested by our results, it is not clear whether mobility reduction in CDMX contributed to a reduction in the propagation of COVID among metro users. [42] showed a clear temporal relation between the accelerated evacuation of subways in Manhattan and the subsequent leveling out of the COVID-19 incidence curve in that area. Manhattanites could afford to stay away from the subway, while many residents of the other four boroughs could not.

Other factors influencing mobility is the risk perception and personal fears of infection that lead to a change in behavior. The authors [43] documented employees’ perceptions of psychosocial safety, because they have to go to their workplaces using buses, subways, or other vehicles, and are under mental pressure for fear of infection, dismissal for non-attendance, and consequent economic problems.

The primary prevention for COVID-19 has been focused on behavioral strategies (e.g., social distancing); however, individuals may have low response trajectories even with some perceived risk. Coifman et al. found that emotions drive the enactment of COVID-19 preventive health behaviors. Both fear and happiness/joy were predictive of approach and avoidance health behaviors [44]. They additionally reported that avoidance behaviors were actions people could perform while staying in their homes (e.g., working from home) and many of the approach behaviors that involve leaving home and greater mobility (e.g., buying cleaning supplies) were not performed. In cities like MAD and NYC where a high percentage of the population belongs to the formal economy sector compared to CDMX, it is expected that adherence to following the recommendations for social distancing will be greater and mobility will decrease.

Existing research that allows for a better understanding of how public transportation impacts mortality due to COVID-19 is very limited. In [45], the relation between daily trips at 496 NYC subway stations and positive cases of COVID-19 was investigated. Data were collected by the New York City Health Department and reported weekly by the Metropolitan Transport Authority. [45] also found a positive trend in the number of positive COVID-19 cases at the beginning of March, followed by a decrease at Easter, and, conjointly, an important reduction in the number of trips on NYC subway systems. Specifically, the reduction was equal to 11.7 positive cases per 10,000 inhabitants, and a concurrent decrease of 10% in the number of daily trips. These findings include all NYC postal codes in the aforementioned period.

Previous research has investigated the impact of mobility reduction as part of the measures implemented for mitigating the COVID-19 pandemic [46]. In other examples, data about mobility, collected through cellphones and anonymized, have been made available by companies such as Google and Apple [47]. Other initiatives, such as Open-SKY, have made data related to flights available. Authors [48] investigated changes in mobility in 25 regions of the United States as a result of the implementation of social distancing policies and their impact on COVID-19 infection rates. This study found that mobility patterns were negatively correlated with COVID-19 positive cases in the most affected regions, with Pearson correlation coefficients greater than 0.7 for 20 of the 25 evaluated regions.

The mobility index proposed by Google has been widely used for measuring how changes in human mobility have flattened the curve of positive cases. This is an open resource and gathers information on mobility for more than 100 countries [49]. When analyzing data about any pandemic, a general approach in epidemiology is to use the SIR model, which is composed of three groups of variables: Susceptible, Infected, and Recovered. These groups might also be non-linear functions with parameters given by mobility reduction interventions. Comorbidities and sociodemographic factors can also be included in the model as covariates. The pandemic is considered “manageable” as long as the number of infected people is lower than healthcare capacity, with the latter being the main objective of reducing human mobility.

A study conducted in Tokyo, Japan evaluated whether reducing human mobility contributed to mitigating COVID-19 positive cases by examining mobility in retail stores, places of recreation, supermarkets, pharmacies, public train stations, offices, and residences, between February 15th, 2020, and July 21st, 2020. The study found differences in human mobility between the first and the second wave. While human mobility dramatically decreased during the first wave, mainly as consequence of the emergence of a new unknown virus, for the second wave a relaxation in the interventions was observed, and therefore a significant reduction in human mobility was not observed [50].

Another aspect to consider in the discussion of these results is the methodology that each investigated city adopted for collecting data on positive cases. An important aspect is that infected people might be asymptomatic. Many of those infected might experience mild symptoms and never request testing. Additionally, there is a significant likelihood that the official numbers in the investigated cities are underestimated. Besides, the estimation of mortality rates in the three cities studied is carried out with certain differences in registration [27, 51, 52].

## Conclusion

The main strength of this study is the generation of results based on observed data, such as subway mobility and mortality rates. Additionally, this paper has proposed a novel CI called *ψ*, which represents the relation between mortality due to COVID-19 and mobility, which might be helpful for governments and policy makers. Through CCF, it is possible to estimate to what extent NPIs, operationalized in mobility reductions, impact mortality due to COVID for the investigated cities. As shown in Fig 2A–2C, the point *t* = 0 at vertical axes represents the day when mobility reduction was introduced and later its effect on ±11 days from left to right. As reported in the results section, there are significant differences between MAD and NYC on the one hand and CDMX on the other.

On the other hand, the main limitation of our study is that the mobility data presented only reflect behaviors of subway use; there is no information about cycling or walking, which has possibly influenced the reduction of mobility in the three cities studied.

Considering that this study is based on mortality records of the cities examined, and these numbers might be underestimated, our results are limited and should be analyzed carefully. Further research based on data that uses estimates of the number of undiagnosed individuals should be conducted, due to issues of low testing rates and cases occurring outside of hospitals.

Moreover, the results presented here are helpful in implementing strategies focused on restricting mobility and reducing the number of deaths due to COVID-19. While reducing the impact of COVID-19 on mortality is the main goal of NPIs, consequences of NPIs on poverty, the economy, unemployment, mental health, crime, and violence should be carefully analyzed in future research. While mitigating the number of deaths is the main purpose of restrictions on mobility, governments and policy makers should consider balanced alternatives to minimize the undesired effects on the economy, unemployment, or mental health. For instance, governments should consider implementing schedules for staggered working hours, limiting the number of passengers per transport unit, providing adequate ventilation in public transport (if possible), and promoting green transport methods such as walking and cycling, among others.

## Supporting information

S1 Dataset Data from official sources on cases, deaths and mobility for each city. (CSV)

S2 Dataset Description dataset. (XLSX)
